# SDhaP: haplotype assembly for diploids and polyploids via semi-definite programming

**DOI:** 10.1186/s12864-015-1408-5

**Published:** 2015-04-03

**Authors:** Shreepriya Das, Haris Vikalo

**Affiliations:** Department of ECE, The University of Texas at Austin, Austin, Austin USA

**Keywords:** Haplotype assembly, Semi-definite programming, Diploid, Polyploid

## Abstract

**Background:**

The goal of haplotype assembly is to infer haplotypes of an individual from a mixture of sequenced chromosome fragments. Limited lengths of paired-end sequencing reads and inserts render haplotype assembly computationally challenging; in fact, most of the problem formulations are known to be NP-hard. Dimensions (and, therefore, difficulty) of the haplotype assembly problems keep increasing as the sequencing technology advances and the length of reads and inserts grow. The computational challenges are even more pronounced in the case of polyploid haplotypes, whose assembly is considerably more difficult than in the case of diploids. Fast, accurate, and scalable methods for haplotype assembly of diploid and polyploid organisms are needed.

**Results:**

We develop a novel framework for diploid/polyploid haplotype assembly from high-throughput sequencing data. The method formulates the haplotype assembly problem as a semi-definite program and exploits its special structure – namely, the low rank of the underlying solution – to solve it rapidly and with high accuracy. The developed framework is applicable to both diploid and polyploid species. The code for SDhaP is freely available at https://sourceforge.net/projects/sdhap.

**Conclusion:**

Extensive benchmarking tests on both real and simulated data show that the proposed algorithms outperform several well-known haplotype assembly methods in terms of either accuracy or speed or both. Useful recommendations for coverages needed to achieve near-optimal solutions are also provided.

## Background

Humans are diploid organisms with two sets of chromosomes – 22 pairs of autosomes and one pair of sex chromosomes. The two chromosomes in a pair of autosomes are homologous, *i.e.*, they have similar DNA sequences and essentially carry the same type of information but are not identical. The most common type of variation between chromosomes in a homologous pair are single nucleotide polymorphisms (SNPs), where a single base differs between the two DNA sequences (*i.e.*, the corresponding *alleles* on the homologous chromosomes are different and hence the individual is heterozygous at that specific locus). *SNP calling* is concerned with determining locations and the type of polymorphisms. Once such single variant sites are determined, *genotype calling* associates a genotype with the individual whose genome is being analyzed. Genotypes, however, provide only the list of unordered pairs of alleles, *i.e.*, genotyping does not associate alleles with specific chromosomes. The complete information about DNA variations in an individual genome is provided by *haplotypes*, the list of alleles at contiguous sites in a region of a single chromosome. Haplotype information is of fundamental importance for a wide range of applications. For instance, when the corresponding genes on homologous chromosomes contain multiple variants, they often exhibit different gene expression patterns. This may affect an individual’s susceptibility to diseases and response to therapeutic drugs, and hence suggests directions for medical and pharmaceutical research [1]. Haplotypes also reveal patterns of variation that are present in certain regions of a genome. This enables focusing whole genome association studies on *tag SNPs* (as in HapMap project [2]), representative SNPs in a region of the genome characterized by strong correlation between alleles (*i.e.*, high linkage disequilibrium). Finally, since each chromosome in a homologous pair is inherited from one of the parents, knowledge of haplotype structure can be used to advance understanding of recombination patterns and identification of genes under positive selection [3].

Haplotypes of an individual whose genome is sequenced can be assembled using short reads obtained by high-throughput sequencing platforms. Each read provides information about the order of nucleotides in a fragment of one chromosome of the individual. Recent advances in high-throughput sequencing allow single individual haplotyping on the whole chromosome level. Previously, the comparatively shorter reads as well as short insert lengths limited the size of connected components. Now, the unprecedented amounts of reads and increasingly longer inserts make haplotyping of an entire chromosome as a single connected block a distinct possibility. In particular, paired-end reads that may be separated by several thousands of bases allow us to link haplotype information over long distances and thus enable their reliable reconstruction. In the absence of any read errors (and errors in alignment and genotyping, i.e., the steps performed prior to haplotyping), haplotype assembly for diploid species is trivial. However, due to the errors in the data processing pipeline steps that precede haplotyping, the assembly is computationally challenging.

Various formulations of the haplotype assembly problem have been proposed [4]. In this paper, we focus on the minimum error correction (MEC) formulation, which attempts to find the smallest number of nucleotides in reads whose flipping to a different value would resolve conflicts among the fragments from the same chromosome. Finding the optimal solution to the MEC formulation of the haplotype assembly problem is known to be NP-hard for the diploid case [4].

### Prior work

Haplotype assembly, also referred to as single individual haplotyping, was first considered in [4] where three related formulations of the problem were described. It has been shown that the problem is computationally hard under various objective functions [4,5]. Levy, 2007 [6] proposed a greedy algorithm for the haplotype assembly of a diploid individual genome. Bansal, 2008 [7] (HapCUT) used a greedy max-cut formulation of the haplotype assembly problem to significantly improve on the performance of [6]. Bansal, 2008 [8] (HASH) and [9] relied on MCMC and Gibbs sampling schemes to tackle the same problem. Wang, 2005 [10] and [11] used computationally intensive branch-and-bound and dynamic programming schemes, respectively, in search for near-optimal solutions to the MEC formulation of the problem. Recently, [12] reformulated the haplotype assembly problem as an integer linear program that was then solved using IBM’s CPLEX. RefHap [13], also relying on a greedy cut approach, was recently introduced and applied to reads sequenced using fosmid libraries while HapCompass [14] relied on a graphical approach to develop a scheme which resolves conflicts arising from incorrect haplotype phasing.

In recent years, genome sequences of polyploid species – characterized by having more than two homologous sets of chromosomes – have been extensively researched. Examples of such organisms include potato (which is tetraploid) and wheat (hexaploid). As in the case of diploid organisms, complete information about genetic variations in polyploid species is provided by their haplotypes. Haplotype assembly for polyploids, however, is significantly more challenging than that for diploid species. Unlike the diploid case, there exist considerably fewer methods for the assembly of polyploid haplotypes. Authors of the first one, [15], addressed the polyploid haplotype assembly by extending the ideas of their HapCompass diploid assembly framework. More recently, [16] (HapTree) investigated the polyploid setup using a branch-and-bound scheme.

### Main contributions

In this work, haplotype assembly is cast as a correlation clustering problem and efficiently solved using a novel algorithm that exploits structural features of the underlying optimization. Correlation clustering, originally proposed by [17] and analyzed in [18-20], is a method for clustering objects that are indirectly described by means of their mutual relationships. In the context of haplotype assembly, the relationships between reads may conveniently be represented by a graph and an associated weighted adjacency matrix, and the problem of assigning reads to haplotypes leads to correlation clustering on this graph. For diploids, that can in principle be done using an algorithm for MAXCUT such as [21], while for polyploids one could use an algorithm for MAX-k-CUT [22]. Both of these algorithms solve semi-definite programming (SDP) relaxations of the original integer programming objectives that arise in MAXCUT and MAX-k-CUT ^a^. The complexity of solving the SDPs, however, is impractical for large-scale haplotype assembly problems. To this end, we develop a novel algorithm for finding low-rank approximate solutions to the aforementioned SDP problems with complexity that is only linear in the number of reads. The results on both simulated and real data sets demonstrate that the proposed algorithm, named SDhaP, has higher accuracy and is significantly faster than the existing haplotype assembly schemes. The proposed method is scalable and needs only minutes to accurately assemble haplotypes of complex genomes on a standard desktop computer. In addition to the developed software, we also provide an in-depth analysis of the coverage required to achieve near-optimal haplotype assembly – a result with many practical implications and useful guidelines for the choice of parameters of sequencing experiments.

## Methods

Haplotype assembly is preceded by the mapping of the reads obtained from a sequencing platform to the reference genome and genotyping. Since homozygous sites do not contribute useful information nor cause any ambiguities in the haplotype assembly, they are omitted from the haplotype and read representations. We represent haplotypes by *K* strings, $(\underline {h}^{1},\underline {h}^{2}, \dots \underline {h}^{K})$, each of length *n*, where *K* denotes the ploidy and *n* is the haplotype length (for diploids, *K*=2). For convenience, each read is represented as a string of length *n* with entries {*A*,*C*,*G*,*T*,−} (denoted by), where ‘ −’ indicates SNP positions on the chromosome that are not covered by the read. The reads are arranged into an *m*×*n* matrix *R* according to their positions along the chromosome, where *m* denotes the number of reads and the *i*^*th*^ row of *R*, *R*_*i*_, corresponds to the *i*^*th*^ read. Since the reads are relatively short compared to the length of the haplotype sequence, matrix *R* is sparse, i.e., a large fraction of its entries are −. The start and the end of the *i*^*th*^ read are the first and the last position in *R*_*i*_ that are not −. The length of a read starting at position *i* and ending at position *j* is equal to *j*−*i*+1 and may include gaps. The goal of haplotyping is to infer $(\underline {h}^{1},\underline {h}^{2}, \dots \underline {h}^{K})$ from the observed reads.

Following genotyping, we identify alleles at each SNP location. Using the genotype calls, one can reduce the underlying alphabet to a ternary one having elements {1,2,−} in the diploid case, and quaternary alphabet {1,2,3,−} in the triploid case. For higher ploidy, there is no further reduction in the alphabet size. In the case where two or more haplotypes share the same nucleotide at a given SNP location (which is not applicable to the diploid setting but can occur for higher ploidy), further reduction is possible by treating all nucleotides that are not a part of the genotype as errors and neglecting them.

### Preprocessing

Before the actual assembly, disconnected haplotype components need to be separated, i.e., we need to identify haplotype blocks that are not connected by any reads. From *R*, we can generate an adjacency matrix and a graph having vertices that correspond to the SNP positions (*i.e.*, to the columns of *R*). An edge is present between two vertices if a read covers the corresponding SNP positions, i.e., if the components of a read at those columns in the matrix are not −. Reads that cover only one SNP position do not provide information that can be used to reconstruct a haplotype and are thus discarded from *R*. Similarly, any SNP position not covered by at least one read is removed. After forming the adjacency matrix, disconnected subgraphs or partitions need to be identified. This is done by implementing a simple queue. Starting with the first vertex, all vertices connected to it are inserted in the queue. These vertices are labeled by *k*=1 to indicate the first subgraph. Then in a first-in first-out manner, all vertices connected to the vertices in the queue are inserted into the queue provided they have not been previously labeled. Once the queue is empty, a new unlabeled vertex is chosen and labeled as *k*=2, and the process is repeated until all vertices are labeled. This procedure leads to partitioning of the matrix into smaller disconnected matrices (if such disconnected components exist).

### Problem definition

Let us define a measure of distance *d* between two symbols *a* and *b* from the alphabet used to represent the SNP fragment matrix *R* as

$$d(a,b)=\left\{\begin{array}{ll} 1 &\text{if} \hspace{1mm} a \neq - \text{and} \hspace{1mm} b \neq - \text{and} \hspace{1mm} a \neq b,\\ 0, &\text{otherwise.} \\ \end{array}\right. $$

Denote the Hamming distance between read *R*_*i*_ and haplotype $\underline {h}^{l}$ as $\text {hd}(R_{i},\underline {h}^{l}) = \sum _{j=1}^{n} d(R_{i,j},\underline {h}_{j}^{l})$. Then the minimum error criterion (MEC) formulation of the haplotype assembly problem is concerned with minimizing *Z* over $\underline {h}^{l}$, where the objective function

(1) $$ {\fontsize{9.1}{6}\begin{aligned} {}Z = \sum_{i=1}^{m}\min\left(\text{hd}\left(R_{i},\underline{h}^{1}\right), \text{hd}\left(R_{i},\underline{h}^{2}\right),\dots \text{hd}\left(R_{i}, \underline{h}^{K}\right)\right), \end{aligned}}   $$

and *m* denotes the total number of reads.

#### The all-heterozygous and heterozygous/homozygous case

Ideally, the SNP matrix *R* should only contain true heterozygous sites determined in the genotyping step. However, in practice, false positives from the genotyping procedure lead to the presence of columns in *R* that correspond to both homozygous sites as well as heterozygous ones. Our method can detect the potential presence of genotyping errors and enable correction of a large fraction of incorrectly called heterozygous sites, hence improving the MEC score of the final solution to the haplotype assembly problem.

#### Problem reformulation

Sequencing reads that are used in haplotype assembly projects may be the short reads generated by Illumina platforms, the long reads obtained from Pacific Biosciences instruments, or the long reads from jumping libraries in [13], to name a few. Consequently, the SNP fragment matrix may be either a fat matrix (with more columns than rows) or a tall one (with more rows than columns), depending on the technology used. While short Illumina paired-end reads generally lead to limited lengths of connected haplotype blocks, technologies that provide long reads and/or large insert sizes enable very long blocks. In the latter scenario, the APX hardness^b^ result essentially implies that exact inference, being of exponential complexity, is no longer feasible. Therefore, computationally efficient approximate inference methods that enable fast yet accurate haplotype assembly are needed.

To quantify the relationships between the reads, we evaluate a measure of similarity for each pair of rows of the SNP fragment matrix as described next. Define a graph $G=(\mathcal {V},\mathcal {E},W)$ where  denotes the set of vertices corresponding to the rows of the SNP fragment matrix,  is the set of the edges connecting the vertices in , and *W* denotes the set of weights associated with the edges. For any two reads *i* and *j* that overlap in at least one position, we define the weight of an edge between the corresponding vertices *v*_*i*_ and *v*_*j*_ as

$$w_{ij}=\frac{k_{sim}-k_{dissim}}{k_{sim}+k_{dissim}}. $$

Here *k*_*sim*_ denotes the number of overlapping positions where the reads have an identical base and *k*_*dissim*_ is the number of positions where they are different. Then $G=(\mathcal {V},\mathcal {E},W)$ is a correlation graph where the edges connecting vertices associated with similar reads (i.e., the reads that belong to the same haplotype) should have positive weights, while the edges connecting vertices associated with dissimilar reads should have negative weights. In the absence of sequencing errors, that is indeed the case and thus separating the reads into *K* different clusters corresponding to *K* distinct haplotypes is trivial. In the presence of errors, however, a positive weight no longer unambiguously implies that two reads belong to the same chromosome nor a negative one means that they belong to different chromosomes, hence making the separation problem difficult. We formalize it as follows: *given a weighted graph*$G=(\mathcal {V},\mathcal {E},W)$, find *K*−1 cuts such that the sum of intra-partition edge weights is maximized and inter-partition edge weights is minimized. This effectively translates to performing ‘correlation clustering’ in machine learning/algorithms parlance.

### Haplotype assembly via correlation clustering

#### Problem formulation for diploid species

In the case of diploid organisms, correlation clustering interpretation of the haplotype assembly problem leads to maximization of the cut norm of the adjacency matrix *W*,

$$ \begin{aligned} & \underset{x}{\text{maximize}} & & \sum_{i<j} w_{ij}x_{i}x_{j} \\ & \text{subject to} & & x_{i} \in \{+1,-1\} \qquad i = 1 \dots m. \end{aligned} $$

Defining $\underline {x} = [x_{1} \; x_{2} \dots x_{m}]^{T}$ where (·)^*T*^ denotes the transpose, the above optimization can be restated as

(2) $$ \begin{aligned} & \underset{\underline{x}}{\text{maximize}} & & \underline{x}^{T}{W}\underline{x}\\ & \text{subject to} & & {x_{i}^{2}}=1 \qquad i = 1 \dots m. \end{aligned}   $$

Introduce a rank-1 matrix variable $X = \underline {x}\underline {x}^{T}$. It is straightforward to show that *X* is positive semidefinite. Thus the maximization (2) can be written as

(3) $$ \begin{aligned} & \underset{X}{\text{maximize}} & & \text{Tr}(WX)\\ & \text{subject to} & & \text{Diag}(X)=\underline{e} \\ & & &\text{rank}(X)=1 \\ & & &X \succeq 0, \\ \end{aligned}   $$

where $\underline {e}$ denotes an *m*×1 all-ones vector. Note that 2 and 3 are equivalent (We omit the details for this). This problem is hard to solve because of the rank constraint. Relaxing the rank constraint leads to the following semi-definite program (SDP),

(4) $$  \begin{aligned} & \underset{X}{\text{maximize}} & & \text{Tr}(WX)\\ & \text{subject to} & & \text{Diag}(X)=\underline{e} \\ & & &X \succeq 0. \\ \end{aligned}  $$

This SDP can efficiently be solved in polynomial-time (in the case of haplotype assembly, *O*(*m*^3.5^) where *m* is the number of reads), and provides an upper bound on the objective of the quadratic program (2). The Goemans-Williamson randomized algorithm may then be used to find an approximate integer solution to the problem [21]. We omit the arguments behind the randomized algorithm for brevity and summarize the procedure below.

Goemans-Williamson algorithm for solving the MAXCUT problem:

- Solve the SDP relaxation and denote the optimal solution by *X*^∗^.
- Compute the factorization *X*^∗^=*VV*^*T*^. Let *V*_*i*_ denote the normalized *i*^*th*^ column of V.
- Rounding Procedure: set $\mathcal {S} =\{\}$.
  - Uniformly generate a random vector $\underline {\eta }$ on the unit *n*-sphere.
  - For *i*=1…*m*, if $x_{i} ={V^{T}_{i}} \eta > 0$ assign vertex *i* to  (i.e., set *x*_*i*_=1); otherwise, assign vertex *i* to $\bar {\mathcal {S}}$ (i.e., set *x*_*i*_=−1).
  - Find the value of the obtained cut $\underline {x}^{T}{W}\underline {x}$.
- Repeat the rounding procedure and output the assignment with best cut value.

#### Problem formulation for polyploid species

In the case of polyploid species, haplotype assembly can be cast as the correlation clustering problem where the goal is to partition the set of reads into as many subsets as there are haplotypes. Let the ploidy of an organism be *K*>2, e.g., *K*=3 for triploids, *K*=4 for tetraploids, and so on. Given the clustering graph $G=(\mathcal {V},\mathcal {E},W)$ representing the reads, we would like to partition the vertex set  into *K* partitions such that the sum of intra-partition edge weights is maximized and inter-partition edge weights is minimized.

To this end, we first need a suitable way of defining variables that can take one of *K* possible values [22]. Let $\underline {y}_{j}$ be one of the *K* vectors {*a*_1_,*a*_2_,…,*a*_*K*_} that are defined as follows: take an equilateral simplex $\sum _{K}$ in $\mathcal {R}^{K}$ with vertices $\{\underline {b}_{1}, \underline {b}_{2},\ldots, \underline {b}_{K}\}$. Let $\underline {c}_{K} = \frac {\underline {b}_{1} + \underline {b}_{2} + \dots + \underline {b}_{K}}{K}$ be the centroid of $\sum _{K}$ and let $\underline {a}_{i} = \underline {b}_{i}-\underline {c}_{K}$, for 1≤*i*≤*K*. Assume that $\sum _{K}$ is scaled so that $\|\underline {a}_{i}\| = 1$ for 1≤*i*≤*K*. Note that this definition of the variables $\underline {y}_{i}$ implies

$$\begin{aligned} &\underline{y}_{i}^{T} \underline{y}_{j} \ge -\frac{1}{K-1} &\qquad i<j \quad i,j=1 \dots m\\ &\underline{y}_{i}^{T}\underline{y}_{i}=1 &\qquad i=1 \dots m. \\ \end{aligned} $$

To see why this is true, note that for any *K*, the entries of $\underline {y_{i}}$ are $-\frac {1}{K}$ except for one of the components which is equal to $1-\frac {1}{K}$. This object is then normalized by its 2-norm and thus (after normalization) $\|\underline {y_{i}}\|^{2} = 1$. When we multiply 2 such normalized vectors, it is straightforward to see that the resulting inner product $\underline {y_{i}}\underline {y_{j}} = -\frac {1}{K-1}$ (*i*≠*j*). Finally, this equality is relaxed to an inequality to turn the problem into a convex problem.

Now we can state the correlation clustering formulation of the haplotype assembly problem for the *K*-ploid species as the optimization

(5) $$  \begin{aligned} & \underset{\underline{y}}{\text{maximize}} & & \sum_{i<j}w_{ij}\underline{y}_{i}^{T} \underline{y}_{j}\\ & \text{subject to} & & \|\underline{y}_{i}\| = 1 \; & i = 1 \dots m \\ & & & \underline{y}_{i}^{T} \underline{y}_{j} \ge -\frac{1}{K-1} \qquad &i,j = 1 \dots m, \quad j<i. \\ \end{aligned}  $$

Define matrix $\hat {Y}$ whose *i*^*th*^ row is $\underline {y}_{i}^{T}$ and introduce $Y = \hat {Y}\hat {Y}^{T}$. The optimization (5) is a vector program which we relax to a semi-definite program of the form

(6) $$ \begin{aligned} & \underset{Y}{\text{maximize}} & & \text{Tr}(WY)\\ & \text{subject to} & & \text{Diag}(Y)=\underline{e} \\ & & &Y_{ij} \ge -\frac{1}{K-1} \qquad i,j = 1 \dots m \\ & & &Y \succeq 0 \\ \end{aligned}   $$

and solve using interior-point methods; here we relaxed the rank of *Y* from *K*−1 to *m*. As in the diploid case, a randomized rounding algorithm may then be used to find an approximate integer solution (details of the rounding procedure are omitted for brevity). Note, however, that there are *m*^2^ constraints which make the complexity of solving the SDP very high, approximately *O*(*m*^7^).

For long haplotype blocks, directly solving the semi-definite programming formulation of the assembly problem in either diploid or polyploid setting is computationally infeasible. It is therefore of interest to explore underlying structural features of the assembly problem and derive fast SDP methods tailored for finding the solution to problems with such features. In the following two sections, we exploit sparsity of the underlying graphical representation of the haplotype assembly problem and the prior knowledge that the solution is low-rank to develop fast yet highly accurate algorithms for solving the SDPs (3) and (6).

### Low-rank SDP solutions for haplotype assembly of diploid species

Barvinok, 1995 [23] and [24] independently studied the scenario where the optimal solution to an SDP has low rank. In particular, they considered the rank-*r* optimal solutions *X*^∗^ to an SDP such that $\frac {r(r + 1)}{2} \le m$ where *m* denotes the number of constraints of the SDP. The existence of *X*^∗^≽0 implies the existence of some $V_{o} \in \mathbb {R}^{m \times r}$ such that $X^{*} = V_{o}{V_{o}^{T}}$. If the optimization over *X*=*VV*^*T*^ is replaced by an equivalent optimization over *V*, the number of variables can be greatly reduced and hence the optimal solution can be found with potentially significant computational savings.

The graph $G=(\mathcal {V},\mathcal {E},W)$ representing the haplotype assembly problem is inherently sparse, and hence we can rewrite (4) as

(7) $$ \begin{aligned} & \underset{V}{\text{maximize}} & & \sum_{i<j} w_{ij}{V_{i}^{T}} V_{j} \\ & \text{subject to} & & \|V_{i}\|=1 \qquad i=1 \dots m, & & & \end{aligned}   $$

where *V*_*i*_ denotes the *i*^*th*^ row of *V*. Note that by expressing the objective function in terms of *V* rather than *X*, we no longer need to explicitly impose the positive semidefinite constraint on *X*. If the graph is very sparse, most *w*_*ij*_’s are 0 and the computation of the objective function is fast. Moreover, it is convenient to convert (7) into the following unconstrained program,

(8) $$  \begin{aligned} & \underset{V}{\text{maximize}} & & \sum w_{ij}\frac{{V_{i}^{T}} V_{j}}{\|V_{i}\| \|V_{j}\|}.\\ \end{aligned}  $$

Denote the objective in (8) by *M*. This optimization is no longer convex; however, for *r*>*r*^∗^ (*r*^∗^ being the rank of the optimal solution), the stationary point of the non-convex problem (8) does in fact coincide with the optimal solution of the convex program (4).

#### Adaptive rank update

To solve (8), we rely on adaptive rank scheme where we initialize *V* as an *m*×2 matrix. In the subsequent steps of the algorithm, the number of columns of *V* is increased until *V* becomes rank deficient (i.e., the rank of *V* drops below the number of columns of *V*). Each step of our proposed scheme requires computation of the objective function (8) and its gradients, which has complexity $O(|\mathcal {E}|r)$. Clearly, we also need to find the rank of *V* as the algorithm progresses. This is done by computing a singular value decomposition (SVD) of *V* (which requires *O*(*mr*^2^) operations) and declaring that the rank of *V* is equal to the number of singular values that are larger than a predefined threshold *ε*_*th*_ (e.g., *ε*_*th*_=0.1).

#### Gradient Computation

We compute the gradient of the objective function in (8) with respect to *V*_*i*_,

$$\begin{aligned} \frac{\partial M}{\partial V_{i}}= \sum_{k \in E_{i}} w_{i,k}\frac{\| V_{i}\|^{2} V_{k}-(V_{i}\cdot V_{k})V_{i}}{\| V_{k} \| \| V_{i} \|^{3}}. \end{aligned} $$

From the computed gradient, we arrive at the following simple update rules for fast iterative solution of the SDP relaxation (8),

$$\begin{aligned} & V_{i} \leftarrow \sum_{k \in \mathcal{E}_{i}} (w_{ik}) V_{k} \\ & V_{i} \leftarrow \frac{V_{i}}{\|V_{i}\|}. \end{aligned} $$

#### Convergence and stopping criterion

We keep track of how the ratio of the gradient to the objective function changes through the iterations. When this ratio becomes smaller than a predefined tolerance value *ε*_*tol*_, we terminate the algorithm. Since the gradient descent scheme ensures that the objective of the optimization problem is non-decreasing, convergence of the algorithm is guaranteed.

#### Randomized projections and greedy refinement

The result of the previously described optimization procedure $\hat V$ is of rank $\hat r$. In order to obtain a rank 1 solution, we project $\hat V$ onto a random vector of size $\hat {r}$ and take the sign of the projection. We generate multiple projections and choose the one among them leading to the largest value of the objective function in (7) as the solution. The number of projections needed for the expected value of the objective function to meet certain performance guarantees is ≈*O*(*log*(*m*)) [25].

In the scenario where there are no genotyping errors, the previously described procedure provides the haplotype pair $(\underline {h}_{1},\underline {h}_{2})$. This solution is further refined by greedily exploring whether sequential alterations of the bases along the haplotype sequences might lead to even lower MEC scores. In the scenario where genotyping errors are present, we use the previously described procedure to partition the reads into 2 clusters. In order to assemble the haplotypes from the partitions, we employ the following strategy: for every SNP location and for each partition, we rely on majority voting to decide on the corresponding haplotype position. This may result in both heterozygous and homozygous sites. Finally, the assembled haplotypes are further greedily refined by testing if sequential alterations of the bases lead to any improvement of the MEC scores, which has complexity *O*(2*n*). We formalize the proposed scheme as Algorithm 1 given below.



### Fast Lagrangian relaxation for haplotype assembly of polyploid species

In the previous section, we described a fast and accurate method for haplotype assembly of diploid species that relies on solving low-rank SDP relaxation of the problem. For the polyploid setting, we need to solve a *constrained* SDP (6). To this end, we employ a fast, low-rank Lagrangian scheme followed by randomized projections and a greedy refinement of the *K*-ploid haplotypes.

Following factorization *Y*=*VV*^*T*^, we can re-phrase the SDP formulation (6) of the haplotype assembly problem for *K*-ploids (5) as the optimization

(9) $$ \begin{aligned} &\!\!\!\!\!\!\!\!\!\! \text{maximize} &&\!\!\!\! \sum_{i<j} w_{ij}\left({V_{i}^{T}} V_{j}\right)\\ &\!\!\!\!\!\!\!\!\!\! \text{subject to} &&\!\!\!\! \|V_{i} \|=1 &&\!\!\!\!\!i=1 \dots m\\ & && {V_{i}^{T}} V_{j} \ge -\frac{1}{K-1} &&\!\!\!\!\!i,j=1 \dots m,\;i<j, \;w_{ij} \neq 0.  \end{aligned}  $$

Unlike the unconstrained optimization (??) that arises in the diploid setting, the above optimization problem is constrained (with conic constraints). In order to solve it with practically feasible and scalable complexity, we consider its Lagrangian relaxation and solve the dual problem using a minorization-maximization technique.

In particular, our scheme iteratively finds

$$\underset{\lambda_{ij} \le 0}\inf \underset{V}\sup \; \mathcal{L}(V,\lambda), $$

where $\mathcal {L}(V,\lambda)$ is the Lagrangian of (9) and *λ*={*λ*_*ij*_} is an *m*×*m* matrix collecting all Lagrange multipliers associated with inequality constraints (the equality constraints need not be explicitly incorporated in $\mathcal {L}(V,\lambda)$ since they are readily enforced by the projection step explained later in this section). Therefore, the Lagrangian is given by

$$\begin{aligned} {}\mathcal{L}(V,\lambda)= &\sum w_{ij}\left({V_{i}^{T}} V_{j}\right) +\sum \lambda_{ij}\left({V_{i}^{T}} V_{j} + \frac{1}{K-1}\right). \\ \end{aligned} $$

The minorize-maximize iterative procedure consists of an inner and an outer loop. In the inner loop (minorize), we find $\underset {V}\sup \textit {L}(V,\lambda)$ by keeping *λ* fixed. For this, we rely on the same idea of cyclic coordinate descent with adaptive rank update as described in the diploid section. Specifically, we make the following updates of the *i*^*th*^ row of *V*,

$$\begin{aligned} & V_{i} \leftarrow \sum_{j \in E_{i}} \left(w_{ij}+\lambda_{ij}\right) V_{j}, \\ & V_{i} \leftarrow \frac{V_{i}}{\|V_{i}\|}. \end{aligned} $$

In the outer loop (maximize) we keep *V* fixed and update *λ*.

The subgradient for *λ*_*ij*_ is ${V_{i}^{T}} V_{j} +\frac {1}{K-1}$. A simple updating rule for *λ*_*ij*_ is of the form

$$\lambda_{ij} \leftarrow \lambda_{ij}+\alpha \left({V_{i}^{T}} V_{j} +\frac{1}{K-1} \right), $$

where *α* is a pre-defined step size. Since *λ*_*ij*_ are constrained to be less than or equal to zero, the above update rule is modified as

$$\lambda_{ij} \leftarrow \min \left(\lambda_{ij}+\alpha\left({V_{i}^{T}} V_{j} +\frac{1}{K-1}\right),0\right). $$

To accelerate the convergance, let us introduce *ε*_*g*_≥0 that defines a guard interval. If *λ*_*ij*_<−*ε*_*g*_, we make a further modification and update *λ*_*ij*_ as

$$\lambda_{ij} \leftarrow \lambda_{ij}2^{-\mu\left({V_{i}^{T}} V_{j} +\frac{1}{K-1}\right)}, $$

where *μ*≥0 is a damping parameter that can be tuned according to the accuracy requirement of the final solution [26]. This exponentiation in the Lagrange multiplier update improves the speed of convergence of the proposed scheme.

#### Convergence and stopping criterion

Detecting convergence is slightly more complicated in the polyploid case as the objective does not increase monotonically. We use a short window of 50 latest iterations to smoothen the value of the objective function and use the obtained value to test the convergence (details omitted for brevity).

#### Randomized projections and greedy refinement

The solution $\hat {V}$ to the fast Lagrangian scheme described in this section has rank $\hat r$. To obtain *K* partitions sought after in the *K*-ploid haplotype assembly problem, we project $\hat {V}$ onto an *r*×*K* matrix *Q* with random entries and assign the *i*^*th*^ read to the *j*^*th*^ partition if the (*i*,*j*) entry of $P = \hat {V}Q$ is the largest component of the *i*^*th*^ row of *P*. We generate multiple projections and choose the one among them leading to the largest value of the objective function in (9) as the solution.

For the no-genotyping-errors setting, the previous scheme provides $\underline {h}^{1},\dots \underline {h}^{K}$. A few rounds of greedy iterations that explore if local alterations of the bases along the *K* haplotype sequences may improve the MEC score are conducted. For the case where genotyping errors are present, we use the previously described procedure to partion the reads into *K* clusters. To assemble the haplotypes from the partitions, we use the majority voting scheme as described in the diploid section. Finally, the assembled haplotypes are further greedily refined by testing if sequential alterations of the bases lead to any improvement of the MEC scores, which has complexity *O*(*Kn*). We formalize the proposed scheme as Algorithm 2 given below.



## Results and discussion

We tested performance of SDhaP using both simulated and experimental data, as described next. Our codes are written in C and the benchmarking tests are conducted on a single core Intel Xeon machine with 2.93GHz and 12GB RAM. We compared SDhaP with CPLEX [12] (an optimal assembly scheme for solving integer programs), HapCUT [7] (a widely used method characterized by a good speed/accuracy trade-off), RefHap [13] (a more recent scheme that is capable of detecting and correcting homozygous positions) and HapTree [16] (a recent method capable of performing haplotype assembly for both diploid and polyploid species).

### Performance on real datasets

#### HuRef Data

We first tested SDhaP on the HuRef dataset [6] which contains single and mated reads sequenced using a dideoxy Sanger sequencing technology with an average coverage of ≈8X. Table 1 compares the accuracy (in terms of the MEC scores) and runtimes of SDhaP with those of aforementioned existing techniques. As can be seen from the table, the MEC scores obtained with SDhaP are significantly better than those of the competing algorithms except for CPLEX. The complexity of CPLEX, however, scales exponentially with the haplotype length which makes it impractical for very long haplotype blocks. As evident from the table, our SDhaP is faster than any of the other considered schemes(except CPLEX). We should point out that unlike SDhaP and RefHap neither HapCUT nor HapTree make homozygous calls, which adversely affects their performance both in terms of MEC and (as shown in the simulation sections) switch error rate (SWER).

**Table 1:** **Comparison of MEC and runtimes for different schemes applied to HuRef data**

**chr #**	**Huref**									
	**CPLEX**		**SDhaP**		**HAPCUT**		**RefHap**		**HapTree**	
	**MEC**	**t(in secs)**	**MEC**	**t(in secs)**	**MEC**	**t(in secs)**	**MEC**	**t(in secs)**	**MEC**	**t(in secs)**
1	16853	1.72×10^5^	17192	190	19777	251	18739	8354	21112	8499
2	12618	1260	12713	220	14698	185	13762	3576	15720	8210
3	9296	960	9444	153	10714	257	10096	4866	11424	12183
4	9958	1140	10106	115	11567	274	10936	5399	12479	9534
5	9195	1080	9333	141	10590	196	10045	4398	11391	8972
6	8637	900	8696	105	9922	247	9318	4225	10912	8462
7	9782	1020	9954	102	11279	152	10540	7030	12196	6876
8	8480	3960	8604	90	9832	226	9352	4640	10552	9162
9	8051	780	8134	88	9290	111	8850	3898	9905	5515
10	8550	1200	8680	87	9877	209	9323	5203	10598	8782
11	7027	840	7186	92	8210	156	7744	4514	8856	7093
12	7136	720	7256	100	8240	152	7725	2669	9003	5494
13	5090	600	5142	62	5844	125	5511	3363	6285	7680
14	5086	480	5173	52	5861	72	5537	2313	6273	3533
15	8088	1.67×10^5^	8216	71	9364	108	9031	2014	10218	4018
16	7176	1500	7231	51	8287	138	7830	1896	8769	4589
17	5739	480	5819	47	6570	86	6238	2362	7106	5626
18	4403	540	4467	56	5041	108	4814	1542	5500	3544
19	4628	480	4670	32	5335	65	5052	1132	5660	2804
20	3243	300	3316	37	3753	63	3458	1472	4068	3602
21	3360	2400	3369	31	3914	63	3752	739	4154	1692
22	3908	7.16×10^5^	3973	32	4539	43	4384	1786	4780	1683

MEC and running times for CPLEX, SDhaP, HAPCUT, RefHap and HapTree algorithms applied to HuRef data. SDhaP is more accurate than all schemes except for CPLEX (which is the only one that requires proprietary software). However, for longer blocks, the complexity of the CPLEX scheme may become very high as evident from chromosomes 1, 15 and 22.

#### Fosmid data

To investigate how SDhaP performs when employed for the assembly of very long haplotype blocks, we tested it on the fosmid dataset analyzed in [13]. Table 2 shows the accuracy and runtime comparison of SDhaP with several competing schemes. As can be seen from the table, the MEC scores of SDhaP are better than those of HapCUT, HapTree and RefHap; its runtimes are comparable to those of RefHap, while HapCUT and HapTree are very slow when the coverage is low and read lengths long (as is the case with the fosmid dataset). Overall, SDhaP seems to be robust with respect to the nature of the dataset, e.g., it is fast, compared to other techniques, regardless whether being applied to HuRef or fosmid datasets.

**Table 2:** **Comparison of MEC and runtimes for different schemes applied to Fosmid data**

**chr #**	**Fosmid**									
	**CPLEX**		**SDhaP**		**RefHap**		**HAPCUT**		**HapTree**	
	**MEC**	**t(in secs)**	**MEC**	**t(in secs)**	**MEC**	**t(in secs)**	**MEC**	**t(in secs)**	**MEC**	**t(in secs)**
1	6889	480	7297	8.07	8051	2.61	9550	600	9676	6501
2	6700	451	7214	7.84	7910	2.65	9661	660	9802	7196
3	5122	420	5588	6.49	6111	2.969	7557	360	7705	4847
4	4072	360	4510	5.41	4880	1.81	6265	540	6500	8392
5	4637	762	5029	6.2	5558	2.15	6919	480	7094	5670
6	5248	471	5674	7.26	6341	2.093	7958	2700	–	–
7	4174	464	4509	5.02	4961	2.07	6062	480	6169	5589
8	4301	347	4785	5.09	5092	2.0	6255	615	6379	8316
9	3974	191	4200	4.7	4591	1.76	5463	376	5513	4465
10	4508	270	4765	5.04	5357	2.52	6445	454	6553	4838
11	3903	150	4165	4.63	4620	2.23	5558	457	5625	5183
12	3907	159	4174	5	4686	2.18	5654	360	5770	5654
13	2669	137	2946	3.0	3155	1.1	3967	291	4029	5367
14	2814	413	2971	3.35	3244	1.89	3978	302	4038	4103
15	2903	138	3029	3.09	3341	1.54	4007	250	4116	3357
16	3844	221	4022	4.84	4438	1.66	5086	570	5142	9683
17	3448	295	3586	3.41	4159	1.86	4743	251	4806	3003
18	2337	288	2555	2.69	2801	1.39	3445	240	3493	2303
19	2707	70	2857	2.78	3406	1.35	3898	180	3953	1984
20	2783	305	2943	3.08	3295	1.72	3810	203	3886	1529
21	1367	72	1452	1.44	1601	1.05	1951	134	1979	1410
22	2422	175	2508	3.21	2876	1.69	3260	118	3307	1351

MEC and running times for CPLEX, SDhaP, HAPCUT, RefHap and HapTree haplotype assembly strategies applied to Fosmid data. SDhaP is more accurate than the other schemes except CPLEX and significantly faster than HapCUT or HapTree. For chromosome 6, HapTree did not complete its run within 48 hours and hence the corresponding entry is missing.

### Performance of the algorithm on simulated data

#### Diploid

To characterize how the accuracy of SDhaP depends upon coverage and haplotype block lengths, we perform tests on simulated data sets. In particular, we generate data sets containing paired-end reads with long inserts that emulate the scenario where long connected haplotype blocks need to be assembled. The SNP rate between two human haploid chromosomes is estimated at 1 in 300 [2]. We generate SNPs by randomly choosing the distance between each pair of adjacent SNPs based on a geometric random variable with parameter *p*_*snp*_ (the SNP rate). To simulate a sequencing process capable of facilitating reconstruction of long haplotype blocks, we randomly generate paired-end reads of length 500 with average insert length of 10,000 bp and standard deviation of 10%; sequencing errors are inserted using realistic error profiles [27] and genotyping is performed using a Bayesian approach [28]. At such read and insert lengths, the generated haplotype blocks are nearly fully connected (99.9*%*).

Accuracy of haplotype assembly is naturally expressed in terms of switch errors – the number of switches (recombination events in the inferred phased haplotypes) that are required to obtain the true haplotype phase. This can be expressed as a rate: the number of switches required divided by the number of opportunities for switch error. While our tests of the performance of SDhaP on real datasets are expressed only in terms of the MEC scores, for the simulated datasets we know the ground truth and therefore characterize the performance of SDhaP in terms of both MEC and switch error rate (SWER). Table 3 compares the MEC, SWER and running times of SDhaP with those of HapCUT, HapTree and RefHap. We make these comparisons for haplotype block lengths of 10^3^ and 10^4^ at coverages of 10, 20 and 30. SDhaP’s MEC score is lower and its SWER is nearly half that of the competing schemes. The running times of SDhaP are at least 10 times lower for haplotype block lengths of 10^4^ (although for block lengths of 10^3^ the difference in running times is not as appreciable). Overall, SDhaP clearly outperforms the other consideredmethods.

**Table 3:** **Comparison of SWER, MEC and runtimes for different schemes on simulated diploid data**

**Data**	**Simulated data**											
	**SDhaP**			**RefHap**			**HAPCUT**			**HapTree**		
	**MEC**	**SWER**	**time**	**MEC**	**SWER**	**time**	**MEC**	**SWER**	**time**	**MEC**	**SWER**	**time**
l 10^3^, c 10	86	0.002	4	123	0.009	8	123	0.009	6	123	0.009	31
l 10^3^, c 20	212	0.001	5	293	0.010	169	303	0.011	8	305	0.006	14
l 10^3^, c 30	300	0.001	7	378	0.007	567	377	0.002	7	378	0.001	14
l 10^4^, c 10	1112	0.003	28	1257	0.008	2341	1354	0.011	282	1354	0.010	34905
l 10^4^, c 20	2088	0.003	36	2659	0.008	36392	2774	0.009	680	2774	0.009	35443
l 10^4^, c 30	3482	0.004	81	4164	0.009	39184	4277	0.010	604	4283	0.009	17002

MEC, SWER and running times (in seconds) for SDhaP, RefHap, HAPCUT and HapTree algorithms for simulated data of different lengths (l) and with different coverages (c). The data contains a fixed 1% fraction of genotyping errors. SDhaP is more accurate in terms of MEC and SWER and faster by almost an order of magnitude compared to other schemes for longer blocks.

Since CPLEX is originally designed to find an optimal solution to the haplotype assembly problem, we compared SDhaP with both the original CPLEX as well as its heuristic variant proposed by [12] for different haplotype block lengths, coverages and error rates. We set a time limit of 24 hours for the optimal scheme to complete the assembly task and, if the optimal scheme did not succeed, ran the heuristic method allowing another 24 hours for the completion of the assembly task. Table 4 and Table 5 show the MEC scores, SWER and runtimes for the considered methods. As can be seen from the tables, CPLEX did not complete the task for block lengths of 10^5^ and most of the block length of 10^4^. For block lengths of 10^3^ and error rates 1%, CPLEX achieves the best MEC scores and SWER but its runtimes are significantly slower than those of SDhaP. For very long blocks and high error rates, neither the optimal CPLEX method nor its heuristic variant provided a solution except in one instance where SDhaP actually performed better (in particular, for the block length 10^4^, error rate 5%, and coverage 10).

**Table 4:** **Comparison of SWER, MEC and runtimes for SDhaP and CPLEX on simulated diploid data with 1% error rate**

**Data**	**Simulated data**								
	**SDhaP**			**CPLEX(optimal)**			**CPLEX(heuristic)**		
	**MEC**	**SWER**	**time**	**MEC**	**SWER**	**time**	**MEC**	**SWER**	**time**
l 10^3^, c 10	100	0.001	1	100	0.001	192	100	0.001	57
l 10^3^, c 20	215	0.001	4	215	0.001	1320	215	0.001	373
l 10^3^, c 30	291	0.001	6	291	0.001	1241	291	0.001	910
l 10^4^, c 10	978	0.008	14	-	-	-	972	0.008	4505
l 10^4^, c 20	2039	0.004	33	-	-	-	2039	0.004	89811
l 10^4^, c 30	2988	0.004	68	-	-	-	-	-	-
l 10^5^, c 10	10356	0.008	324	-	-	-	-	-	-
l 10^5^, c 20	19975	0.007	713	-	-	-	-	-	-
l 10^5^, c 30	29967	0.005	1810	-	-	-	-	-	-

MEC, SWER and running times (in seconds) for SDhaP, RefHap, HAPCUT and HapTree algorithms for simulated data of different lengths (l) and with different coverages (c). The data contains a fixed 1% fraction of genotyping errors. SDhaP is more accurate in terms of MEC and SWER and faster by almost an order of magnitude compared to other schemes for longer blocks.

**Table 5:** **Comparison of SWER, MEC and runtimes for SDhaP and CPLEX on simulated diploid data with 5% error rate**

**Data**	**Simulated data**								
	**SDhaP**			**CPLEX(optimal)**			**CPLEX(heuristic)**		
	**MEC**	**SWER**	**time**	**MEC**	**SWER**	**time**	**MEC**	**SWER**	**time**
l 10^3^, c 10	535	0.04	1	518	0.036	811	-	-	-
l 10^3^, c 20	1042	0.007	4	-	-	-	1041	0.007	11393
l 10^3^, c 30	1583	0.003	4	-	-	-	-	-	-
l 10^4^, c 10	4971	0.099	14	4945	0.093	13800	7024	0.12	961
l 10^4^, c 20	9839	0.0370	41	-	-	-	-	-	-
l 10^4^, c 30	15310	0.0150	85	-	-	-	-	-	-
l 10^5^, c 10	51342	0.210	375	-	-	-	-	-	-
l 10^5^, c 20	102234	0.120	772	-	-	-	-	-	-
l 10^5^, c 30	157234	0.030	1517	-	-	-	-	-	-

MEC, SWER and running times (in seconds) for SDhaP, RefHap, HAPCUT and HapTree algorithms for simulated data of different lengths (l) and with different coverages (c). The data contains a fixed 5% fraction of genotyping errors. SDhaP is more accurate in terms of MEC and SWER and faster by almost an order of magnitude compared to other schemes for longer blocks.

**Figure 1:**
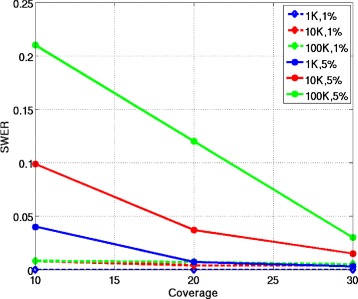
SWER for diploids. Switch error rates of SDhaP applied to diploid data as a function of coverage for different block lengths and error rates. To achieve the same SWER, higher coverages are needed for longer blocks and higher error rates.

Figure 1 shows the switch error rate of SDhaP as a function of sequencing coverage for various block lengths and error rates. The considered haplotype block lengths are 10^3^, 10^4^ and 10^5^. The data for the haplotype blocks is generated with embedded error rates of ≈1*%* and ≈5*%*. The coverages used were 10*X*, 20*X* and 30*X*. As can be seen in the figure, when the error rate is 1%, the SWER of SDhaP for coverages greater than 20*X* is very small for all block lengths. When the error rate is 5%, we observe that higher coverage is needed to ensure low SWER.

**Figure 2:**
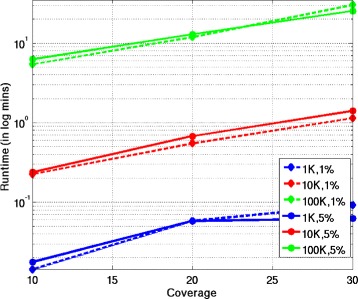
Runtimes for diploids. Runtimes of SDhaP applied to diploid data as a function of coverage for different block lengths and error rates. The runtimes are nearly independent of error rates and scale approximately linearly with block lengths.

Figure 2 show the runtimes of SDhaP as a function of the coverages for various block lengths and error rates. The runtimes (in minutes) are plotted on the logarithmic scale and show that the complexity of SDhaP scales approximately linearly with block lengths and coverage. Notably, a block of length 10^5^ (which is of the same order as the length of the haplotype associated with the longest human chromosome, chromosome 1) with a coverage of 30*X* requires only 20 minutes. Note that there is no significant difference in the runtimes for different error rates.

#### Polyploid

**Figure 3:**
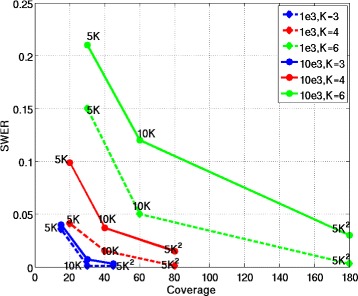
SWER for polyploids. Switch error rates of SDhaP applied to polyploid data as a function of coverage for different block lengths and error rates. To achieve the same SWER, higher coverages are needed for longer blocks and higher ploidy.

**Figure 4:**
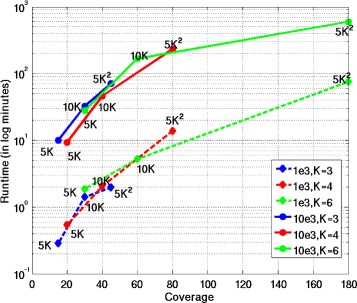
Runtimes for polyploids. Runtimes of SDhaP applied to polyploid data as a function of coverage for different block lengths and error rates. The runtimes scale approximately linearly with block lengths and quadratically with the ploidy.

To test the performance of SDhaP for the assembly of polyploid haplotypes, we generate data in the same way as described in the previous section (notably, the reads and inserts are of the same lengths as those in the diploid simulations). We study the performance of SDhaP when applied to the assembly of haplotypes with ploidy *K*=3, 4 and 6. Figure 3 shows the SWER of SDhaP as a function of the coverage for various block lengths. As can be seen there, the coverage required to obtain a chosen target SWER increases with the ploidy. (For details on the definition of SWER for polyploids, please see [16]; we compute SWER using a branch and bound scheme). The algorithm is tested for coverages 5*KX*, 10*KX* and 5*K*^2^*X*, where *K* denotes the ploidy. From the simulation results, it appears that the required coverage increases approximately with the square of the ploidy. For example, the coverage needed to achieve SWER below 1% for triploids (*K*=3) is approximately 45*X*, for tetraploids (*K*=4) the required coverage is around 80*X*, and for hexaploids (*K*=6) the algorithm requires coverage of ≈180*X*. In Figure 4 we show the runtimes of SDhaP (in minutes and on a logarithmic scale) as a function of the coverage for different ploidy.

Tables 6, 7 and 8 compare the MEC, SWER and runtimes of SDhaP when applied to the haplotype assembly of triploids, tetraploids and hexaploids as a function of coverage and block length with those of HapTree [16]. Note that HapTree, previously shown to outperform the only other existing method for haplotype assembly of polyploids [16], assumes exact knowledge of the underlying genotypes and that its performance deteriorates in the presence of errors. Genotyping from next-generation sequencing data, however, is typically erroneous [29] and hence we compare the performance of SDhaP and HapTree in the presence of genotyping errors (the error rates, reported in the tables, are typical of genotyping software [29]). As can be seen from the tables, SDhaP outperforms HapTree in terms of both SWER and MEC. The complexity of SDhaP is roughly linear in the size of the haplotype block while the complexity of HapTree grows significantly with the size of the block. In fact, several of HapTree simulations could not be completed within 48hrs (hence the data for such instances is missing).

**Table 6:** **Comparison of SWER, MEC and runtimes for different schemes on simulated biallelic triploid data**

**Dataset parameters**	**Genotyping error rate**	**SDhaP**			**HapTree**		
		**MEC**	**SWER**	**t(in secs)**	**MEC**	**SWER**	**t(in secs)**
length 10^3^, cov 15	0.0348	170	0.0130	18	401	0.0430	1860
length 10^3^, cov 30	0.0135	331	0.0027	90	582	0.0220	8
length 10^3^, cov 45	0.0064	488	0.0013	183	614	0.0053	5
length 10^4^, cov 15	0.0348	1848	0.0143	388	-	-	-
length 10^4^, cov 30	0.0135	4091	0.0038	1289	4744	0.0191	680
length 10^4^, cov 45	0.0064	6169	0.0025	2048	5492	0.0060	2424

MEC, SWER and running times for SDhaP and HapTree algorithms on biallelic triploid simulated data. For *l* = 10^4^ and c = 15, HapTree did not complete the task in 48 hrs.

**Table 7:** **Comparison of SWER, MEC and runtimes for different schemes on simulated biallelic tetraploid data**

**Dataset parameters**	**genotyping error rate**	**SDhaP**			**HapTree**		
		**MEC**	**SWER**	**t(in secs)**	**MEC**	**SWER**	**t(in secs)**
length 10^3^, cov 20	0.0487	193	0.0105	40	626	0.0891	580
length 10^3^, cov 40	0.0217	385	0.0050	124	974	0.0380	780
length 10^3^, cov 80	0.0081	836	0.0015	560	2174	0.0290	15
length 10^4^, cov 20	0.0487	4676	0.0233	383	-	-	-
length 10^4^, cov 40	0.0217	6966	0.0096	2901	-	-	-
length 10^4^, cov 80	0.0081	14146	0.0072	8784	-	-	-

MEC, SWER and running times for SDhaP and HapTree algorithms on biallelic tetraploid simulated data. For *l* = 10^4^, HapTree did not complete the task in 48 hrs.

**Table 8:** **SWER, MEC and runtimes of SDhaP for simulated hexaploid data**

**Dataset parameters**	**Genotyping error rate**	**SDhaP**		
		**MEC**	**SWER**	**t(in secs)**
length 10^3^, cov 30	0.0480	1270	0.1338	278
length 10^3^, cov 60	0.0283	1653	0.0215	943
length 10^3^, cov 120	0.0177	2246	0.0170	1178
length 10^3^, cov 180	0.0087	1767	0.0017	8341
length 10^4^, cov 30	0.0480	14127	0.3370	1665
length 10^4^, cov 60	0.0283	16014	0.1100	5240
length 10^4^, cov 120	0.0177	21102	0.0353	19940
length 10^4^, cov 180	0.0087	72203	0.0210	30911

MEC, SWER and running times of SDhaP for biallelic hexaploid simulated data. HapTree completed the task within 48 hrs in only one case, (*l* = 30, cov=60), where it achieved MEC=2832, SWER=0.1114, and *t* = 3441 s, all inferior compared to the results of SDhaP in the table.

Remark: SDhaP is designed to minimize the MEC score which, as pointed out in [16], cannot distinguish between identical pairs of SNPs on the haplotypes of a polyploid. For example, when a triploid has pairs of SNPs {AC,GT,GT} at the same positions of its haplotypes, MEC cannot be used to distinguish between the two chromosomes containing the SNP subsequence GT (and thus phase the corresponding haplotypes). However, this does not impede the ability of the MEC criterion to enable separation of polyploid haplotypes provided they are sampled by paired-end reads sufficiently long to resolve segments of identical SNPs – as demonstrated by the results presented in Tables 4, 5 and 6.

### Connectivity

**Figure 5:**
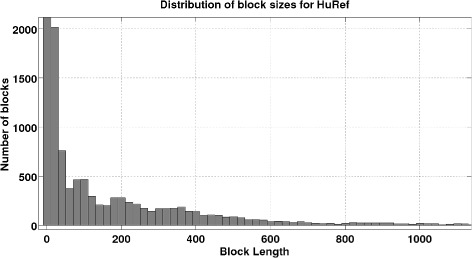
Block lengths histogram for HuRef data. Histogram of block lengths for Huref data.

**Figure 6:**
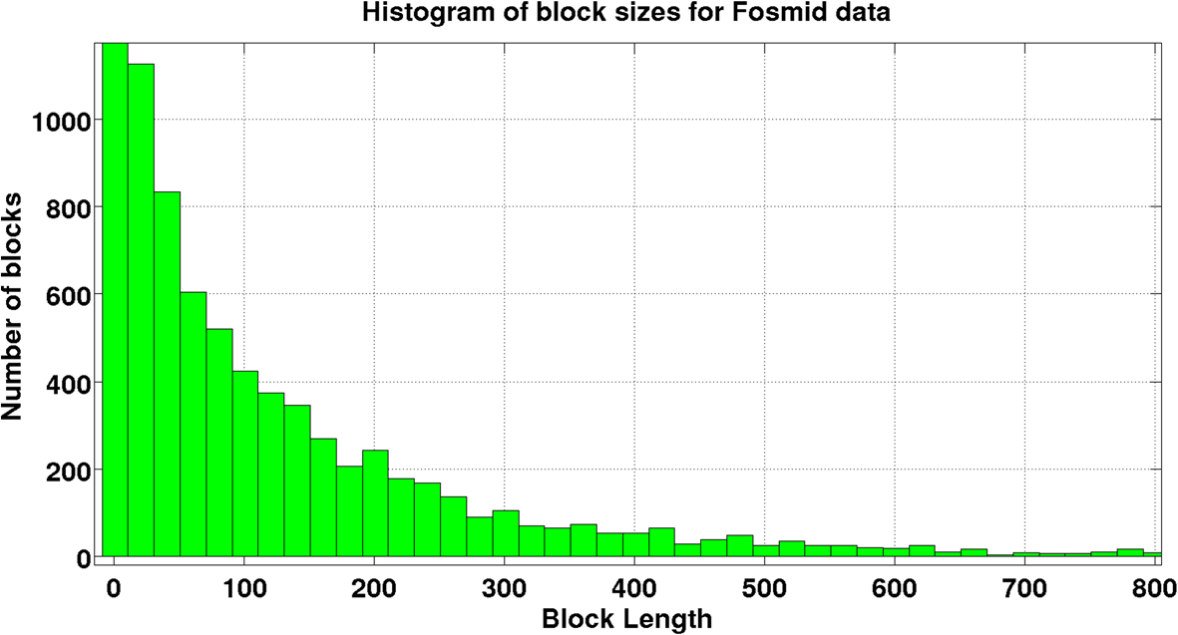
Block lengths histogram for fosmid data. Histogram of block lengths for Fosmid data.

The lengths of sequencing reads, insert sizes and their variations, and SNP rates are of fundamental importance for the achievable performance of haplotype assembly and connectivity of SNP positions. Figure 5 and Figure 6 show the distributions of the haplotype block lengths for HuRef and Fosmid data for all 22 chromosomes, respectively. As can be seen there, majority of the blocks are shorter than 500 SNPs. While this has been a major issue with previous generations of sequencing technologies, with the ability of sequencing longer reads and fosmid technologies that allow insert lengths as large as 100kB, one can expect achieving complete connectivity in future. In our simulation studies, we focused on long inserts that enable near-complete connectivity of the haplotype blocks. For a more detailed discussion, please see [30] and the references therein.

### Homozygous positions

**Figure 7:**
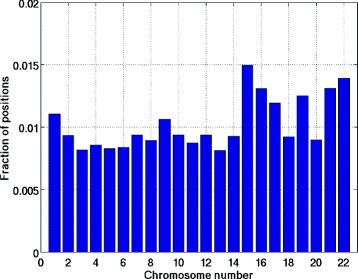
Histogram of homozygous positions. Histogram of the fraction of homozygous positions as a function of chromosome number for HuRef data. On average, around 1% positions are falsely called heterozygous.

Chen, 2013 [12] demonstrated presence of homozygous sites in haplotype blocks assembled using high-throughput sequencing data. In Figure 7, we show the histogram of the fraction of homozygous positions in the haplotypes assembled from HuRef data using SDhaP. As seen there, approximately 1−1.5*%* of the positions are homozygous. To address this issue, [14] suggested using alternative measures of performance such as minimum weighted edge removal (MWER). However, as our results demonstrate, optimizing the MEC objective with the added capability of calling homozygous positions results in a very low SWER.

## Conclusion

In this paper, we introduced a haplotype assembly scheme for diploid (*K*=2) and polyploid (*K*>2) species that relies on our novel technique for solving low-rank semidefinite programming optimization problems. Highly accurate and computationally efficient, the proposed SDhaP algorithm also addresses the important issue of having homozygous positions in the data – a problem that is neglected by most existing haplotyping schemes. The method is tested on real and simulated data for both the diploid and polyploid scenarios, showing that it outperforms several existing methods in terms of both accuracy and speed. We also provide important guidelines for the required coverage needed to achieve near-optimal haplotype assembly. In future, we expect to extend the developed method to jointly perform genotyping and haplotyping.

## Endnotes

^a^ In semidefinite programming, one minimizes a linear function subject to the constraint that an affine combination of symmetric matrices is positive semidefinite. Such a constraint is nonlinear and nonsmooth, but convex, so semidefinite programs are convex optimization problems. Semidefinite programming unifies several standard problems (e.g. linear and quadratic programming) and finds many applications in engineering and combinatorial optimization [31].

^b^ In complexity theory, the class APX is the set of NP optimization problems that allow polynomial-time approximation algorithms with approximation ratio bounded by a constant (or constant-factor approximation algorithms for short).
